# Editorial: Airways and malocclusion: etiology and treatment outcomes

**DOI:** 10.3389/froh.2025.1733643

**Published:** 2025-12-05

**Authors:** Michele Tepedino, Mauro Lorusso, Domenico Ciavarella

**Affiliations:** 1 Department of Clinical and Experimental Medicine, University of Foggia, Foggia, Italy; 2 Department of Biotechnological and Applied Clinical Sciences, Dental School of L'Aquila, University of L'Aquila, L'Aquila, Italy

**Keywords:** malocclusion, orthodontic, airway obstruction, craniofacial growth and development, sleep-related breathing disorders

## Abstract

The correlation between the upper airway and malocclusion is still a controversial issue. Craniofacial abnormalities and dental malocclusions, such as mandibular retrusion, maxillary constriction, and vertical skeletal discrepancies, are strongly associated with reduced airway patency and increased pharyngeal collapsibility. This relationship underscores the importance of evaluating craniofacial morphology in both the diagnosis and management of OSAS. Treatment aims not only to reduce apneic events and improve sleep quality but also to restore normal breathing dynamics and prevent long-term cardiovascular, metabolic, and neurocognitive complications. Achieving these goals requires a multidisciplinary approach that integrates orthodontics, surgery, otolaryngology, and sleep medicine to provide effective and long-term outcomes.

## Introduction

The craniofacial morphology may have an important role in upper airway anatomy. The reduction of the upper airway space may increase the risk of Obstructive Sleep Apnea start. Obstructive sleep apnea syndrome (OSAS) has increasingly been recognized as a highly prevalent and multifactorial disorder with significant clinical and socioeconomic implications. In Europe, it affects an estimated 44.3 million adults, posing a substantial clinical and socioeconomic burden (1). The pathogenesis reflects the interplay of anatomical predispositions that narrow the upper airway and the sleep-related decline in dilator muscle tone, particularly of the genioglossus, which together promote pharyngeal collapse (2). Affecting both pediatric and adult populations, OSAS is associated with cardiovascular and metabolic morbidity, impaired neurocognitive development, behavioral disturbances, reduced quality of life, and increased healthcare costs. The heterogeneous nature of this condition requires a personalized, multidisciplinary approach that draws upon the expertise of pediatricians, otolaryngologists, orthodontists, pulmonologists, radiologists, and maxillofacial surgeons.

## Special issue evidence

This Special Issue brings together a total of nine articles, including two corrigenda, reflecting the breadth and diversity of current research in the field. The contributions stem from international teams based in China, Italy, Switzerland, the United States, and Thailand, underscoring the global relevance of obstructive sleep apnea research. The articles, published between March 2023 and September 2025, collectively provide a timely and comprehensive overview of diagnostic and therapeutic advances across different age groups and clinical settings.

Two articles focus specifically on the pediatric population. Wei et al. explored the application of drug- induced sleep endoscopy (DISE) in infants with dynamic upper airway collapse. The study demonstrates how DISE, by reproducing sleep conditions pharmacologically, provides real-time visualization of airway dynamics. This technique allows clinicians to localize the precise site and degree of obstruction, which is particularly crucial in very young patients where traditional assessments are limited by age and cooperation. Complementing this, Shi et al. investigated the diagnostic value of morphological data derived from volumetric computed tomography, integrated with clinical indices. The results suggest that quantitative imaging parameters, such as airway volume, cross-sectional area, and craniofacial characteristics significantly enhance diagnostic accuracy when combined with clinical evaluation. Importantly, this approach can help distinguish children who are likely to benefit from surgical interventions, such as adenotonsillectomy, from those requiring alternative or adjunctive therapies.

The pediatric section of this Special Issue also addresses therapeutic strategies, combining critical reflection with long-term clinical evidence. Rinchuse et al. examined the role of the orthodontist in pediatric OSAS. The authors emphasize that while orthodontic interventions such as rapid maxillary expansion or mandibular repositioning appliances hold promise for improving airway dimensions, the evidence supporting their effectiveness remains limited. Authors argue that orthodontics must not be practiced in isolation but rather integrated within an interdisciplinary framework that includes pediatricians, otolaryngologists, and sleep specialists. Sriboonyong et al. provided a compelling 20-year experience with CPAP administered via tracheostomy in children with tracheomalacia. This rare and severe condition is associated with recurrent airway collapse and life-threatening events. The authors demonstrate that long-term CPAP, even when delivered through tracheostomy, can significantly improve survival, growth, and neurodevelopmental outcomes. The complexity of chronic tracheostomy care is evident, involving substantial psychosocial and logistical challenges for families.

Finally, Guerin et al. presented a corrigendum to a case report on long-term non-invasive ventilation (NIV) in an infant with Hallermann–Streiff syndrome. The correction highlights the importance of precision in reporting but also reiterates a key clinical message: syndromic patients often require highly personalized, prolonged ventilatory strategies. The case underscores the necessity of tailoring interventions to genetic and craniofacial anomalies, reinforcing the heterogeneity of pediatric OSAS and the need for flexible, individualized management approaches.

### Orthodontic and surgical approaches in adults

In the adult population, three articles explore innovative therapeutic strategies. Ciavarella et al. investigated sleep position shifts in patients with OSA treated with mandibular advancement device (MAD). The study expands the current understanding of MAD, traditionally evaluated for the ability to maintain upper airway patency. MAD therapy may also influence positional behavior during sleep, suggesting a broader mechanism of action that integrates mechanical and behavioral effects. This finding opens new avenues for evaluating oral appliances not only in terms of respiratory indices but also in their impact on sleep architecture. A corrigendum (Ciavarella et al.) was later published, ensuring transparency and accuracy in reporting.

Cretella Lombardo et al. compared twin-block appliances with mandibular advancement achieved through clear aligners, a modality gaining increasing popularity in orthodontics. The comparative study is highly relevant, as it explores both incremental and maximal mandibular advancement strategies. The results provide novel insights into how different appliance designs affect airway volume, helping clinicians balance therapeutic efficacy with patient comfort and long-term dental stability.

Li et al. offered an anatomic and aerodynamic assessment of a modified maxillomandibular advancement (MMA) procedure in East Asian patients with moderate to severe OSAS. The study recognizes that craniofacial morphology varies significantly across ethnic groups and that surgical approaches must be adapted accordingly. By tailoring MMA to East Asian anatomical characteristics, authors demonstrated improved airway outcomes and reduced pharyngeal collapsibility.

Although addressing different aspects of the disorder, the articles in this Special Issue converge on a central message: the management of OSAS must be individualized, grounded in an accurate assessment of each patient's anatomical, functional, and clinical characteristics. Diagnostic innovations, ranging from DISE to volumetric imaging, are opening new avenues for more targeted interventions. At the same time, the diversity of therapeutic options, from orthodontics and mandibular advancement to surgery and long-term ventilatory support, highlights the need for effective multidisciplinary integration.

## Conclusion

This Special Issue offers a comprehensive perspective on the evolving landscape of obstructive sleep apnea management across age groups and clinical settings. Taken together, the articles highlight that OSAS cannot be managed through a uniform strategy; instead, effective care requires individualized approaches informed by each patient's anatomical, functional, and clinical characteristics. Advances in diagnostic techniques are opening new avenues for earlier and more accurate patient identification and treatment planning. At the same time, the broad range of therapeutic strategies, which includes orthodontics, mandibular advancement devices, surgical innovations, and long-term ventilatory support, underscores the essential role of multidisciplinary collaboration.
